# Natural Hydrogen Sulfide Donors from *Allium* sp. as a Nutraceutical Approach in Type 2 Diabetes Prevention and Therapy

**DOI:** 10.3390/nu11071581

**Published:** 2019-07-12

**Authors:** Sonia Melino, Sara Leo, Vilma Toska Papajani

**Affiliations:** 1 Department of Chemical Science and Technologies, University of Rome “Tor Vergata”, via della Ricerca Scientifica 1, 00133 Rome, Italy; 2 CIMER Center for Regenerative Medicine, University of Rome Tor Vergata, via Montpellier 1, 00166 Rome, Italy; 3 Department of Pharmacy, University of Medicine Tirana, rruga Dibra, 371 Tirana, Albania

**Keywords:** OSCs, garlic, phytochemicals, inflammation, oxidative stress, H_2_S, diabetes, plants, nutraceuticals

## Abstract

Type 2 diabetes mellitus (DM) is a socially relevant chronic disease with high prevalence worldwide. DM may lead to several vascular, macrovascular, and microvascular complications (cerebrovascular, coronary artery, and peripheral arterial diseases, retinopathy, neuropathy, and nephropathy), often accelerating the progression of atherosclerosis. Dietary therapy is generally considered to be the first step in the treatment of diabetic patients. Among the current therapeutic options, such as insulin therapy and hypoglycemic drugs, in recent years, attention has been shifting to the effects and properties—that are still not completely known—of medicinal plants as valid and inexpensive therapeutic supports with limited side effects. In this review, we report the relevant effects of medicinal plants and nutraceuticals in diabetes. In particular, we paid attention to the organosulfur compounds (OSCs) present in plant extracts that due to their antioxidant, hypoglycemic, anti-inflammatory, and immunomodulatory effects, can contribute as cardioprotective agents in type 2 DM. OSCs derived from garlic (*Allium* sp.), due to their properties, can represent a valuable support to the diet in type 2 DM, as outlined in this manuscript based on both in vitro and in vivo studies. Moreover, a relevant characteristic of garlic OSCs is their ability to produce the gasotransmitter H_2_S, and many of their effects can be explained by this property. Indeed, in recent years, several studies have demonstrated the relevant effects of endogenous and exogenous H_2_S in human DM, including by in vitro and in vivo experiments and clinical trials; therefore, here, we summarize the effects and the underlying molecular mechanisms of H_2_S and natural H_2_S donors.

## 1. Introduction

Diabetes mellitus (DM), as reported in the WHO 2016 global report, is a chronic disease with high incidence worldwide, creating a crucial social issue that represents one of four major noncommunicable diseases as outlined in world forums. The International Diabetes Federation recently estimated that DM affects about 425 million people and that this number will increase to 629 million in 2045. Type 2 DM is the most frequent form of the disease (over 90% of DM patients), characterized by hyperglycemia due to insulin resistance or inadequate insulin secretion [1]. Progressive hyperglycemia is one of the main causes of oxidative stress and is recognized to be principally responsible for type 2 DM complications [2,3,4]. Inflammation and oxidative stress are determinants for the loss of endothelial function and dysfunction of the vascular endothelium leading to macrovascular (cerebrovascular or heart pathologies), as well as at the microvascular complications (degenerative defects of the kidney or retina, with subsequent complications such as limb amputation or neurological defects) [5,6]. Dementia, depression, sexual dysfunction, and high risk for cancers of the liver, pancreas, colon, and rectum are other complications stemming from chronic diabetic conditions [7]. Epidemiological evidence suggests that type 2 DM is frequently under-diagnosed according to a recent review in seven countries, which estimated that 24% to 62% of people with DM were undiagnosed and untreated [8]. The main causes of type 2 DM are related to genetic factors and lifestyle (patterns of diet and physical activity) [9]. Obesity and physical inactivity are known, by epidemiological evidence, to be risk factors for insulin resistance and type 2 DM. Accordingly, several studies have examined the effect of a combination of diet and physical activity, often referred to as a lifestyle intervention, in reducing the progression of Impaired Glucose Tolerance to type 2 DM [10,11,12,13,14,15]. The U.S. Diabetes Prevention Program (DPP), Finnish Diabetes Prevention Study (FDPS), and Da-Qing Investigation have produced evidence that the risk of developing type 2 DM can be reduced by changes in one’s lifestyle. In both the FDPS and DPP studies, the estimated risk reduction was about 58% after three years [15].

To achieve good and long-term metabolic control in DM, to reduce its complications, and to maintain quality of life, a combination of changes in lifestyle and pharmacological treatment is required. According to both the European Association for the Study of Diabetes (EASD) and the American Diabetes Association (ADA), the lifestyle changes, including Medical Nutrition Therapy (MNT), physical exercise, smoking cessation, and weight loss, are important approaches in the management of type 2 DM [16]. In recent years, there have been a great number of hypoglycemic drugs available for the treatment of type 2 DM, mainly by oral administration, which possess different mechanisms of action. These include, for example, decreasing endogenous glucose production, insulin secretagogue, alpha-glucosidase inhibitor, dipeptidyl peptidase-4 inhibitor, and sodium glucose co-transporter-2 inhibitor 1. Metformin still remains the major frontline drug for the treatment of type 2 DM [17]. Many available drugs for the treatment of DM have significant adverse effects and do not prevent its complications. The high prevalence of type 2 DM and its multiple complications highlight the requirement for further investigations aiming for the improvement of existing anti-diabetic therapeutic regimens or for the development of a new therapeutic strategy based on the current understanding of the pathophysiology and biochemical pathways of insulin resistance. In this context, natural products are a very important source of bioactive compounds acting on distinct molecular mechanisms able to affect several biochemical pathways, providing benefits in diabetic management as part of complementary and alternative therapies or as important new lead molecules for drug design [18,19].

Dietary therapy is generally considered to be the first step in the prevention and treatment of diabetic patients. Among the current therapeutic options, such as insulin therapy and hypoglycemic drugs, attention in recent years has been shifting to the effects and properties—still not completely known—of medicinal plants as valid and inexpensive therapeutic supports lacking or almost completely devoid of side effects.

## 2. Therapeutic Potential of Nutraceuticals Consumed in Type 2 DM

Plants and plant extracts have been used for the treatment of DM since ancient times, and they still remain an important source of herbal remedies in DM therapy, and possible tools for the development of new drugs [20].

The antihyperglycemic biguanide metformin was developed from investigation of the plant *Galega officinalis,* traditionally used to treat DM [21,22]. Herbal remedies are very popular, particularly in developing countries, and play a supportive role as a complementary medical intervention with limited toxic effects and reduced financial cost. Over 400 plants and their compounds have been studied for type 2 DM treatment, and several reviews summarize these studies [23,24,25,26]. According to efficacy, the most active plants in the management of DM are *Trigonella foenum greacum*, *Momordica charantia*, *Gymnema sylvestre*, *Ocimum tenuiflorum*, *Panax ginseng* and *quinquefolius*, *Coccinia grandis*, *Opuntia* spp., *Allium* spp., etc. [26,27,28]. Many studies in human and animal models of type 2 DM have confirmed the potential beneficial effects of plants to correct the metabolic disorder and to delay the development of diabetic complications. However, the therapeutic efficacy of herbal plants in mitigating the deleterious effect of DM remains insufficient (and, in some cases, controversial) to actively recommend the use of herbal medicine to treat either high blood glucose or other related risk factors [20,29]. Table 1 summarizes the effects of the most important plants and their active compounds, for which there is clinical evidence of their efficacy as nutraceuticals or food supplements in the prevention or cure of diabetes. In general, the antidiabetic activity of these plants is attributed to the presence of bioactive compounds, such as polyphenols, terpenoids, alkaloids, coumarins, and other constituents, which have demonstrated a reduction in blood glucose levels. The most common hypoglycemic mechanisms of action found for these plant extracts and their pure compounds include the reduction of α-glucosidase activity, inhibition of protein tyrosine phosphatase 1β and antioxidant activity, activation of the peroxisome proliferator-activated receptors (PPARs), reduction of glucose uptake and glucose transport, and induction of pancreatic insulin secretion [25,30]. The synergistic effect of different phytochemicals in the plant extracts is very important, so that the herbs have multiple mechanisms in the control of the diabetic condition and its complications. In some cases, they lower the blood glucose steady-state level, and they also reduce hypertension and the blood lipid profile [31]. Many plant preparations and derived compounds are used as nutraceuticals or food supplements to prevent DM or as an adjuvant in combined therapy with antidiabetic drugs to treat DM and its complications. Frequently, clinical evidence has demonstrated that supplementary treatment of diabetic subjects with functional foods and nutraceuticals derived from vegetables could increase the effectiveness of DM management [32]. Most nutraceuticals are dietary phytochemicals, such as polyphenols compounds (phenolic acids, flavonoids and their derivatives, stilbenes, tannins), glycosinolates, phytoestrogen, dietary fibers, and carotenoids. Dietary polyphenols possess several biological and beneficial properties and are considered an important class of antioxidant with a beneficial role in opposing the effects of excess reactive oxygen species involved in the pathogenesis of type 2 DM [33,34]. Most epidemiological papers connect dietary polyphenol consumption to reduced risk of type 2 DM [35,36]. Many studies evidentiate that dietary polyphenolic compounds may exert hypoglycemic effects in multiple ways, such as by inhibiting intestinal carbohydrate hydrolyzing enzymes α-amylase and α-glucosidase, reducing intestinal absorption of dietary carbohydrate, protecting β-cell function from glucotoxicity, activating 5-adenosine monophosphate-activated protein kinase (AMPK), increasing insulin-dependent glucose uptake, or showing antioxidative and anti-inflammatory properties [34,37,38,39,40,41]. Phenolic-rich extracts, anthocyanins, and isoflavones have shown protective effects on pancreatic β cells against oxidative damage through enhancing the natural antioxidant system [42,43,44,45]. Flavonoids have been found to lower glucose levels, mainly through inhibiting intestinal α-glucosidase and α-amylase [46,47], upregulating the liver glucokinase (GK) via PPARγ, upregulating adipocyte Glucose transporter-4 (GLUT4) [48,49], inhibiting intestinal glucose absorption by inhibiting GLUT2 [50], or through reduction or decrease the lipid peroxidation [51]. Furthermore, proanthocyanidin extracts from grape seeds have drawn great interest as natural treatments for DM and some long-term DM complications. According to clinical studies, these extracts seem to delay the development of retinopathy, nephropathy, and neurodegenerative damage in diabetic subjects [52,53]. Other studies indicate that epigallocatechin-3-gallate (ECGC) from green tea may act on glucose intestinal and cellular uptake, on inflammation to inhibit adipocyte proliferation, and on oxidative stress [54,55,56,57,58]. ECGCs suppress apoptosis via several mechanisms, including the activation of the nuclear factor erythroid 2-related factor 2 (Nrf2), resulting in subsequent enhancement of expression of the antioxidant response elements (ARE), providing more resistance to reactive oxygen species (ROS) damage via neutralizing enzymes and ROS scavengers. However, for the prevention of type 2 diabetes and obesity, it is important to have slow absorption of the green tea ECGCs, which could be obtained using polyethylene glycol-3350 or poly-γ-glutamate to extend their intestinal effects [57,59]. Therefore, additional trials are needed to support green tea consumption for DM therapy, with larger sample size and greater statistical power.

## 3. OSCs from Garlic as Nutraceuticals for Prevention and Therapy in Type 2 DM 

**Figure 1:**
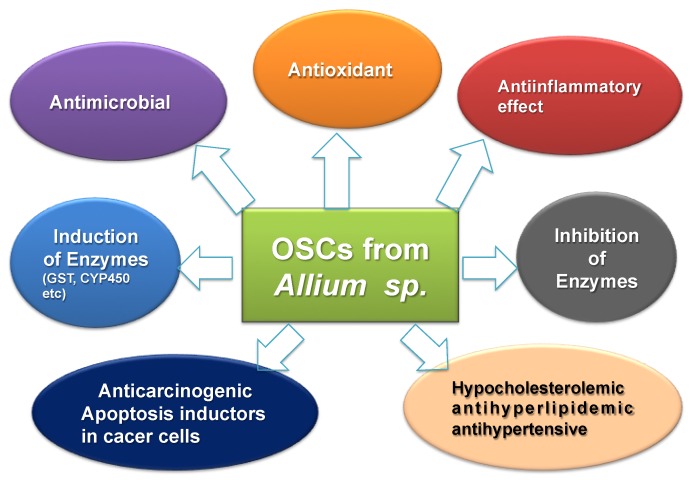
Scheme of the effects of organosulfur compounds (OSCs) derived from *Allium sp*.

Among the nutraceuticals described above, the plant extracts with organosulfur compounds (OSCs) deserve particular interest. Several studies have shown that OSCs and their different formulations inhibit insulin resistance and hyperglycemia, and they subsequently protect DM patients from several clinical effects, including cardiovascular complications. There are two main groups of vegetables characterized by the presence of OSCs with special properties: Brassicaceae and Amaryllidaceae. The first family includes cabbage, cauliflower, and Brussels sprouts, and kale and rucola (also known as rocket salad) are part of the Eruca genus of the mustard or cruciferous family; all of these produce S-methyl cysteine-l-sulfoxide [113]. The second one includes shallot, garlic, leek, onion, and chives; they belong to the Allium genus and produce S-alk(en)yl-l-cysteine sulfoxides. OSCs, contained in both these vegetable families, can be used as nutraceuticals and the mechanisms of action of either original produced sulfoxides or their derivatives have been studied in detail for their therapeutic effects. According to these investigations, type 2 DM patients eating broccoli sprouts, containing sulforaphane (1-isothiocyanato-4-(methylsulfinyl)butane), show increased total antioxidant capacity in their blood, serum insulin, and insulin resistance, with reduced lipid peroxidation, serum triglycerides, oxidative stress index, oxidized low-density lipoprotein (LDL)/LDL cholesterol ratio, and blood high-sensitivity C-reactive protein (CRP) [114]. Therefore, sulforaphane seems to reduce nephropathy, diabetic fibrosis, and vascular complications. The underlying molecular mechanism of sulforaphane seems to involve the Nrf2-related antioxidant response, elevation of phase 2 enzymes and PPARs, reduction of oxidative stress, and NF-κB (nuclear factor kappa light chain enhancer of activated B cells) activity reduction (with reduction of its related inflammation). According to these investigations, sulforaphane, as a component of young broccoli sprouts, is an excellent food additive for diabetic patients [114]. One of the most important glycemic-controlled herbal medicines with OSCs is garlic (*A. sativum* L.) [115,116]. Epidemiological and preclinical studies support the effects of garlic extract and its OSCs as cardiovascular-protective agents [117,118,119,120,121,122,123,124,125,126,127,128,129,130,131,132,133], due to the properties of these compounds, which are summarized in Figure 1. 

**Figure 2:**
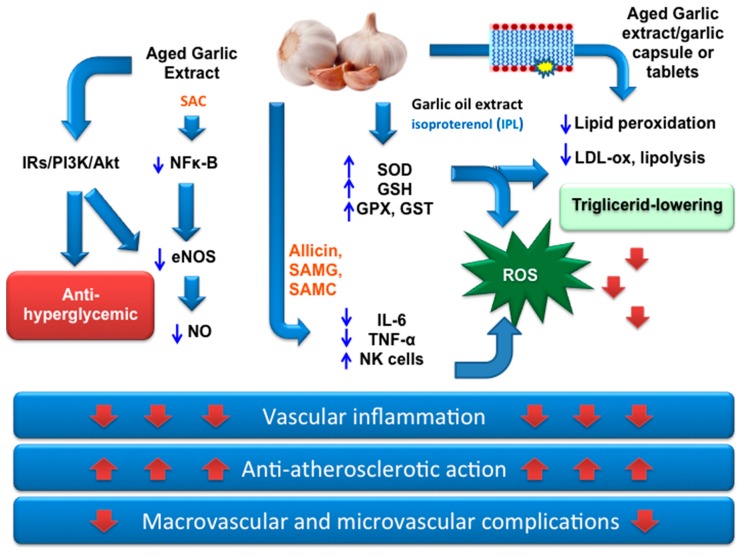
Scheme of the inter-relationship between hyperglycemia, iperlipidemia, oxidative stress, vascular inflammation, and the ability of garlic extract to modulate macrovascular and microvascular complications in type 2 DM [139,149,150,151]. Abbreviations: DM = Diabetes mellitus; P13k/Akt = phosphoinositide-3-kinase/Protein Kinase B; IRs = Insulin Receptors; SAC = S-allyl cysteine; allicin = dyallil thiosulfinate; SAMG = S-allylmercaptoglutatione; SAMC = S-allylmercaptocysteine; NO = Nitric oxide; IL-6 = Interleukin 6; TNF-α = Tumor necrosis factor; NK cells, Natural killer cells; GST = Glutathione-S-transferase; GSH = Glutathione reduced; SOD = Superoxide dismutase; GPx = Glutathione peroxidase; eNOS = endothelial Nitric oxide synthase.

Garlic shows powerful effects in DM, such as hypoglycemia, hyperinsulinemia, hypotriglyceridemia, anti-glycation, hypocholesterolemia, and anti-lipidperoxidation effects [116,117] (Figure 2). Garlic, either dried or fresh, and its derivatives show antihyperglycemic effects in genetic animal models of DM [116,134,135,136,137] and clinically in humans [138,139]. Garlic improves insulin sensitivity and the associated metabolic syndrome in animal models [134], and its derivatives reduce both insulin resistance [67] and blood glucose in streptozotocin-induced and alloxan-induced DM mellitus in rats and mice [140,141]. These beneficial effects are attributed to the presence of OSCs, such as derivatives from alliin and sulfoxide amino acids. The effect of garlic homogenate in reducing heart hypertrophy and fructose-induced myocardial oxidative stress is due to activation of the PI3K/Akt/Nrf2-Keap1-dependent pathway [142]. Diabetic erectile dysfunction may be associated with an elevated level of ROS in penile tissue [143] and ROS formation prevention and the restoration of the erectile function by S-allyl cysteine (SAC), the main OSC in aged garlic extract, in diabetic rats was obtained by modulation of NADPH oxidase expression. Recent studies on SAC demonstrated its antidiabetic, antioxidant, anti-inflammatory, and neuroprotective properties [144,145]. Trials using raw garlic on type 2 DM patients have reported a significant lowering of glycaemia and lipid metabolism with a concomitant amelioration of redox metabolism (SOD, catalase, and GPx in erythrocytes) [146]. Similar effects have been reported by other trials following administration of garlic or garlic compounds [147,148]. Although several investigations using garlic or its extracts, both in animal models and in clinical trials, have shown a clear beneficial effect in the treatment of patients with DM, nonetheless, additional investigations are needed to further explore the benefits of garlic for patients with type 2 DM.

**Figure 3:**
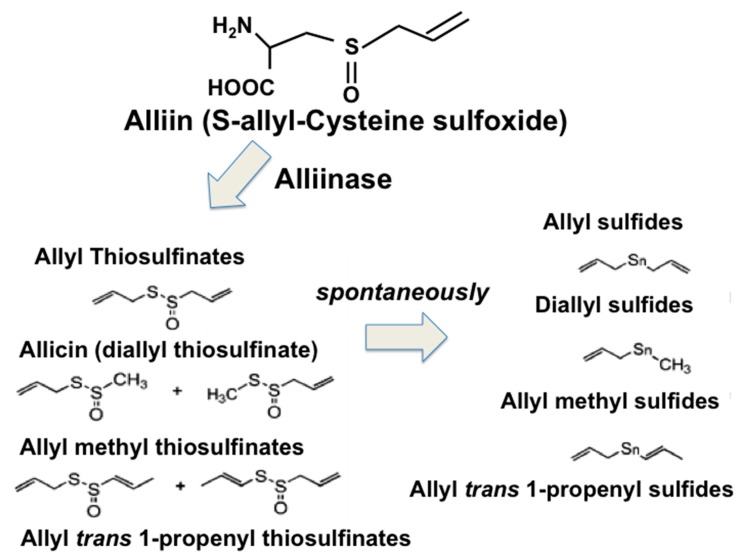
Scheme of the spontaneous OSCs production from garlic.

Garlic OSCs are spontaneously derived from allicin after cutting of the garlic cloves (Figure 3) and are the principal active ingredients that are responsible for the beneficial effects of the garlic extracts. The alliin (S-allyl-cysteine sulfoxide) is metabolized to allicin (diallyl thiosulfinate) by alliinase, a carbon-sulfur lyase enzyme that can be released only by breaking the garlic cells. Subsequently, allicin rapidly undergoes nonenzymatic decomposition that transforms into a series of OSCs, such as diallyl monosulfide (DAS) and oil-soluble polysulfides, including diallyl disulfide (DADS) as a main product and diallyl trisulfide (DATS) (Figure 3).

These first compounds are responsible for the characteristic pungency of garlic [152]. Among the compounds with notable current interest, there are the so-called polysulfites, which are abundant constituents, especially in the essential oils of garlic. Zhao and colleagues [153] identified 16 compounds as the main components of commercial garlic essential oil, accounting for 97.44% of the total oil of *A. sativum*. These were diallyl trisulfide (DATS; 50.43%), diallyl disulfide (DADS; 25.30%), diallyl sulfide (DAS; 6.25%), diallyl tetrasulfide (DATES; 4.03%), 1,2-dithiolane (3.12%), allyl methyl disulfide (3.07%), 1,3-dithiane (2.12%), and allyl methyl trisulfide (2.08%).

Originally, the antidiabetic properties of allicin were demonstrated in rabbits by a reduction of fasting blood glucose, with comparable efficacy to the standard drug tolbutamide [154]. The heart-related complications in DM, such as suppression of myocardial fibrosis progression in streptozotocin-induced diabetic rats, can be reduced by allicin administration. The attenuation of apoptosis and fibrosis after allicin treatment was related to the inhibition of Bcl-2, CD95, connective tissue growth factor (CTGF), and transforming growth factor β1 (TGF-β1) protein expression, eventually preventing DM-induced cardiac complication progression [155]. Bcl-2 and CD95 drive the cell fate, while CTGF and TGF-β1 are highly sensitive myocardial fibrosis markers. Allicin substantially down-regulates both Bcl2 and CD95 in diabetic rats, and thereby reverses myocardial apoptosis remodeling [155]. Moreover, ventricular arrhythmias activated by Bcl-2 treatment in diabetic rats can be considerably suppressed by allicin. Electrophysiology experiments have also demonstrated that allicin attenuated the action potential duration by inhibiting the L-type calcium current (ICa-L) and improving the inward rectifier potassium current (IK1) [156]. Nephropathy is also a disease linked to DM and it is typically related to a high kidney weight/body ratio, blood urea, and creatinine, with a reduced creatinine clearance rate. Allicin treatment efficiently ameliorated the diabetic nephropathy in rats by preventing the effects on the TGF-1/p-ERK1/2 signaling pathway [157].

In order to ameliorate the efficacy and stability of allicin, it was conjugated with captopril to produce S-allyl-mercapto-captopril (CPSSA). Prolonged CPSSA administration reduces body weight gain, blood pressure, and blood glucose levels in Cohen- Rosenthal Diabetic Hypertensive mice. 

All these data demonstrate that allicin can provide an important contribution to reducing obesity, hypertension, and diabetes, which are also important risk factors for cardiac and metabolic disease [158]. Therefore, allicin can prevent insulin resistance and other complications [159]. The molecular mechanism by which allicin reduces the pathologies related to the DM was also investigated, and it was related to its ability to scavenge ROS. Free radical generation, which is also due to hyperglycemia, could in fact be one of the primary causes of insulin resistance in DM and its related complications [160].

In vitro studies have demonstrated that allicin attenuated nicotinamide adenine dinucleotide phosphate oxidase (NOX) activation and ROS production when oxidized LDL-cholesterol was exposed to endothelial cells [159,161]. 

Other derivatives from allicin have been studied for their properties as adjuvants in diabetic pathologies. One of these derivatives is allyl methyl sulfide (AMS), which is one of the major bioactive components in garlic present in the volatile garlic fraction with antibacterial [162], antioxidant [163], and anticancer properties [164]. The administration of AMS to experimental hyperglycemic rats considerably enhanced glutathione (GSH) and vitamin C and E levels [165]. Moreover, AMS treatment, by way of its free radical scavenging property and control of free radicals in the liver, is able to increase the activities of antioxidant enzymes. 

Antioxidants and anti-inflammatory phytochemicals have a crucial role in the prevention of acute liver damage [166]. Due to the hepatoprotective effects of AMS, its administration can reduce the elevation of hepatic injury enzymes [165]. Several studies have shown that AMS treatment improves hepatic cellular damage, thereby conquering diabetic complications. Beneficial effects in alleviating diabetic liver damage and improving the hepatic function were obtained, in addition to exertion of a better glycemic control through stimulating insulin secretion in the remnant β-cells and ameliorating inflammatory markers. Dietary administration of AMS exhibited significant preservation of the structural and functional integrity of hepatocytes, probably due to the attenuation of hyperglycemia-mediated oxidative stress [165]. Further in vivo and clinical studies are necessary to confirm the possibility of using this phytochemical for dietary treatment in DM.

**Figure 4:**
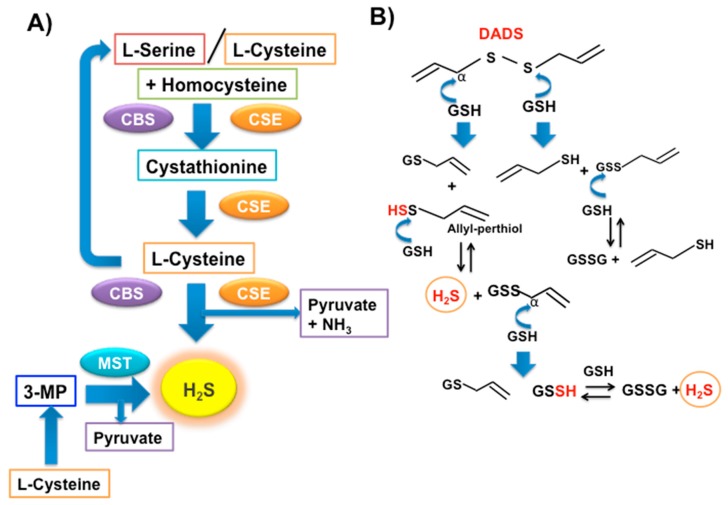
Scheme of the enzymatic (**A**) and non-enzymatic (**B**) production of H_2_S in mammalian cells. The figure B displays the non-enzymatic production of H_2_S starting from DADS that reacts with GSH through a nucleophilic substitution at the α-carbon. Abbreviations: CBS = cystathionine β-synthase; CSE = cystathionine γ-lyase; 3-MP = 3-mercaptopyruvate; GSH = reduced glutathione; GSSG = oxidized glutathione; GSSH = glutathione persulfide; 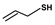 = allyl-thiol; 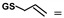 = S-allyl-glutathione; 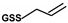 = allyl-glutathione disulfide.

Garlic OSCs and their conjugates are also optimal slow H_2_S-releasing agents [167,168,169] and are able to release H_2_S in a non-enzymatic reaction with intracellular GSH (Figure 4B) [169]. A growing body of evidence has shown that H_2_S plays an important role in the disordered glucose metabolism [170,171] that is the most important features of DM. Therefore, garlic-derived OSC supplementation could increase H_2_S levels, help to restore kidney function, and represent a natural therapeutic strategy. 

## 4. H_2_S-Releasing Agents for Prevention and a Therapeutic Approach in Type 2 DM

**Figure 5:**
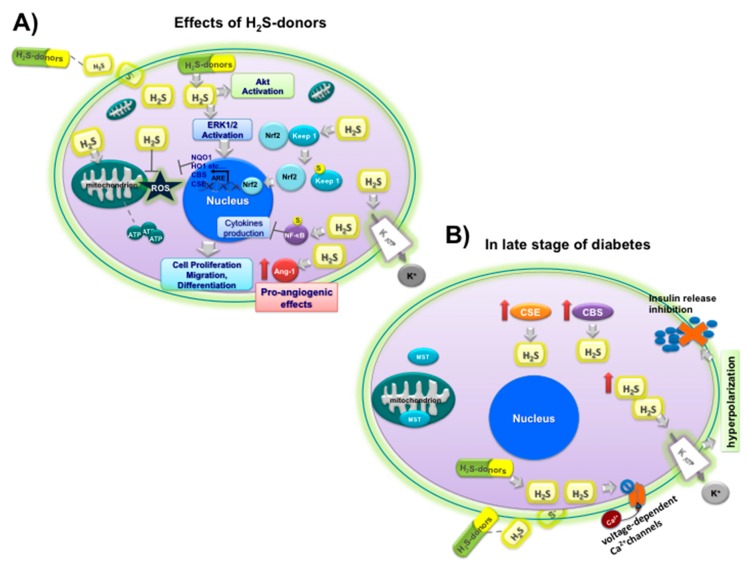
(**A**) Scheme of the effects and pathway activation of H_2_S-donors in cell: Akt activation, Erk1/2 activation, 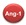 Ang-1 upregulation, 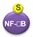 NF-kB sulfidration, 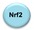 Nrf2 activation by sulfidration of Keep1 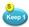 and upregulation of CBS, CSE and antioxidant enzyme (NQO1, HO1, etc.), opening 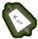 K_ATP_ channels; (**B)** effects of H_2_S on insulin 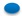 release under hyperglycemic conditions (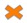 inhibition) at late stage of diabetes in beta cells: upregulation of CSE 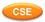 and CBS 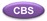; MST 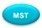; closure 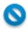 of L-type voltage-dependent Ca^2^C channels 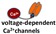, opening of K_ATP_ channels 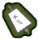, and hyperpolarization.

Hydrogen sulfide is one of three important endogenous *gasotransmitters* and is released in tissues from the metabolism of L-cysteine or polysulfides [170] (Figure 4A,B). Principally, the enzymatic production of H_2_S in mammalian cells is due to the cytosolic pyridoxal 5’-phosphate (PLP)-dependent enzymes cystathionine β-synthase (CBS) and cystathionine γ-lyase (CSE) and to the 3-mercaptopyruvate sulfurtransferase (MST) that is present in both the cytosol and mitochondria. H_2_S exerts relevant protective effects and has essential roles in the central nervous, respiratory, and cardiovascular systems. H_2_S is a physiological mediator able to limit inflammation and free radical damage by reacting with multiple oxidant stressors, including peroxynitrite [172], superoxide radical anion [173], and hydrogen peroxide [174], and by producing glutathione persulfide (GSSH) in mitochondria [175,176,177], a more efficient H_2_O_2_ scavenging molecule than GSH. Its antioxidant activity is also due to activation of the Nrf2-ARE pathway [178] (Figure 5A). In the last few years, H_2_S donors have shown great therapeutic potential for widely diffused pathologies, such as cardiovascular [179,180,181], neurodegenerative [182,183,184], and gastrointestinal diseases [185,186]. Moreover, H_2_S seems to be able to protect islet beta cells from damage elicited by distinct toxic or stress events [187,188]. Both exogenous administration of H_2_S (NaHS) and stimulating endogenous H_2_S generation with L-cysteine seem to reduce programmed cell death [187]. The pathological loss of beta cells, but also the chronic inflammation of damaged islet cells, is the primary cause of several DM complications. DM can be, therefore, considered an inflammatory response disease, suggesting a possible anti-inflammatory therapy [189,190,191]. Of note is that the NaHS administration can significantly inhibit pro-inflammatory-factor-induced injury in primary cultured pancreatic beta cells and MIN6 cells [192]. The reduction of H_2_S by knockout of CSE in high-fat-diet-induced type 2 DM mice exhacerbates oxidative insults without insulin secretion or reduction of blood glucose levels [193]. Actually, endogenous H_2_S does not always work as a friend. Conversely, other authors demonstrated that H_2_S contributes to ER stress-mediated cellular apoptosis through activation of the p38 MAPK pathway [194], not fully in keeping with the work by Taniguchi et al. [192].

Basal CSE expression is quite low in islet β cells, but can be increased by high concentrations of glucose. H_2_S can affect insulin-secreting β cells, both inhibiting secretion of insulin from the cells [195,196] and protecting them against cellular apoptosis induced by various stimuli [193,197]. Deregulated production of insulin is the major reason for glycometabolism disorder, and therefore DM; this is because insulin is the only hormone that is able to decrease blood glucose. Some studies have demonstrated that the expression of CSE and CBS is significantly upregulated in both liver and pancreas in streptozotocin-induced diabetic rats compared to the control [198]. H_2_S administration to beta cell lines INS-1E and HIT-T15 cells attenuated insulin secretion triggered by a high concentration of glucose [195]. This inhibitory effect of H_2_S on insulin secretion is related to the opening of K_ATP_ channels [199]. During diabetic hyperglycemia, high levels of endogenous H_2_S can open K_ATP_ channels in islet β cell membrane, causing elevated polarization of the membrane potential and lower insulin secretion [195] (Figure 5B). Moreover, exogenous H_2_S by NaHS inhibits L-type voltage-dependent Ca^2^C channels, further lowering insulin secretion in a K_ATP_ channel-independent manner [196]. Therefore, H_2_S inhibits insulin secretion by targeting several biochemical processes, such as activation of K_ATP_ channels, inhibition of ATP synthesis, and inactivation of L-type voltage-dependent Ca^2^C channels [170]. Of note is that the physiological synthesis of H_2_S inhibition in Zucker DM rats increased insulin release and reduced hyperglycemia [200]. Altogether, the above strongly support that the downregulation of the H_2_S system apparently promotes diabetic prevention and treatment. The regulation of endogenous H_2_S production can be very relevant in DM, i.e., at the early phases of diabetes, the administration of H_2_S may be beneficial, while at its late stage, inhibiting H_2_S generation may be a possible therapeutic strategy.

On these bases, we can conclude that the effects of H_2_S on insulin secretion can change at different phases of diabetic development. Therefore, in the early phases of the disease, hyperglycemia-induced CSE upregulation seems a beneficial mechanism for the patients and the increased H_2_S levels protect islet β cells by reducing oxidation and inflammation, and by inhibiting the autoimmune response. The development of diabetes leads to a further increase of H_2_S that can inhibit insulin secretion and reduce the overload of diabetic beta cells by the reduction of the ATP content, activation of K_ATP_ channels, or inhibition of L-type voltage-dependent calcium channels [201]. In persistent hyperglycemia conditions, an increase in endogenous H_2_S can trigger an ER stress response, and consequently apoptosis [194]. Although endogenous H_2_S production could have different effects in the stages of DM, several studies have demonstrated that the treatment with H_2_S-releasing agents can be important in reducing the damage induced by DM. Oxidative stress in DM leads to excessive autophagy with consequent vascular endothelial cell (EC) dysfunction. Several studies have shown that exogenous H_2_S administration is able to prevent arterial EC dysfunction by inhibition of excessive autophagy through the Nrf2-ROS-AMPK signaling pathway [202]. NaHS treatment ameliorated myocardial autophagy, and more generally, the myocardial fibrosis, which is a predominant pathological characteristic of diabetic myocardial damage, by PI3K/Akt1 pathway activation [203]. High blood glucose levels and DM are implicated in neurodegeneration, and one of the hallmarks of this pathology is protein aggregation; H_2_S treatment could represent a novel strategy against protein aggregation in the diabetic brain [204]. Other common complications of DM are reduced angiogenesis and intractable wound lesions. H_2_S has been reported to have pro-angiogenic effects and H_2_S donors are able to promote diabetic wound healing by restoring endothelial progenitor cell (EPC) function and inducing an upregulation of in-wound skin tissue and EPCs [205]. In diabetic skin complications, H_2_S provided by NAC or NSHD-1, a synthetic slow H_2_S-releasing donor, can exert protective effects against DM-like injury [206,207]. More recently, several groups have produced slow H_2_S-releasing materials able to promote cell proliferation and migration and tissue repair, also reducing oxidative stress due to ROS [208,209]. A microparticle system comprising hydrophobic phase-change materials able to release H_2_S, termed NaHS@MPs, was produced for wound healing applications in DM [210]. In this study, significantly accelerated re-epithelialization and wound closure in diabetic mice was obtained using Tegaderm integrated with NaHS@MPs. Other H_2_S-releasing biomaterials for potential application in wound healing in DM have been obtained using OSCs derived from garlic. Wang et al. [211] demonstrated that mesoporous silica nanoparticles (MSNs) loaded with DATS, named DATS-MSN, and able to release H_2_S can stimulate endothelial cells proliferation and migration and have cytoprotective effects, reducing the inflammatory cytokines production and adhesion molecule expression. Other formulations of slow H_2_S-releasing microfibrous mats were produced by functionalization or doping with OSCs or oil-soluble extracts derived from garlic, named DADS-PFM/PFM+DADS and GaOS-PFM/ PFM+GaOS [212], and were shown to scavenge hydrogen peroxide, increasing pro-cell survival signaling, and at the same time, decreasing pro-apoptotic signaling. The development of slow H_2_S-releasing biomaterials opens new perspectives for applications of OSC H_2_S-releasing donors for the fabrication of biomedical devices. Functionalized biomaterials could then be used inside or outside the body for both non-implantable devices and patches for wound dressing and implantable vascular grafts and implants in order to reduce damage due to DM, improving the patient’s health. 

## 5. Conclusions

Currently the worldwide attention is focused on the development of prevention and treatment of diseases by daily consume of nutraceuticals, which can have a supportive role in preserving the life quality of the public. In this review we revised the state of the art on the use of nutraceuticals for prevention and adjuvant therapy of type 2 DM and its complications, focusing our attention in particular on nutraceuticals with sulfur and derived from *Allium spp.* The peculiarity of these nutraceuticals is their ability to release the *gasotrasmitter* H_2_S. Although endogenous H_2_S, as a signaling molecule, can show different effects at different stages of DM, several in vitro and in vivo studies have demonstrated that H_2_S donors can reduce the onset of DM and the damage it causes. However, more clinical studies are requested to support the validity of OSCs administration in the prevention and therapy of DM. In general, although several studied have demonstrated the beneficial effects of nutraceuticals in DM, one of the most important problems with natural compounds, including garlic OSCs, is their stability over time. Indeed, many garlic OSCs, such as allicin and its derivatives, can rapidly degrade even at low temperatures. Accordingly, several groups are developing promising strategies of administration of these natural compounds, such as capsules containing garlic oil self-nanoemulsifying systems [213] or nano-emulsions obtained in combination with other nutraceuticals, as we previously have shown with omega 3 and proteins [214], for improving their stability and bioavailability. Therefore, the production of new formulations with other nutraceuticals, having synergistic effects, may be relevant to obtaining good administration and reproducibility in clinical trials. Moreover, the variability of the chemical composition of the vegetables, which can vary with the environmental conditions and countries where they are produced, represents another relevant problem. Therefore, trials on the use of vegetables containing H_2_S donors should include information on their chemical composition and standardized preparations. In this context, the possibility to increase the production of the optimal H_2_S-releasing agents in the OSC-rich-vegetables should be explored in order to produce optimized food for daily usage as a prevention strategy and adjuvant cure for type 2 DM. 

The study of OSCs and the vegetables containing them represents a stimulating field of research, in which the redox biology, inflammation, detoxification, tissue repair, and regeneration are interconnected for beneficial effects on human health.

## Figures and Tables

**Table 1:** The most relevant plants and vegetables and their phytochemicals/nutraceuticals with significant effects on type 2 DM via clinical or in vivo studies.

Plants/Vegetables Species	Phytochemicals/Nutraceuticals	Effects on Type 2 DM	References
*Aegle marmelos* (Common name: bael)	coumarins (umbelliferone β-D-galactopyranoside) alkaloids, and steroids	↓ PPBG and lipid peroxidation; ↑ hypoglycemic effect of standard oral drugs in type 2 DM patients and antioxidant activity	[60,61,62]
*Allium cepa* and *A. sativum.* (Common names: onion and garlic)	OSCs and flavonoids (quercetin and its glycosides)	↓ FBG and intestinal glucosidase inhibition, serum cholesterol and triacylglycerol and LDL-cholesterol; ↓ blood glucose and lipid levels; ↑ GLUT-4 translocation, glucose uptake and insulin action, SOD, GPx and catalase activity	[63,64,65,66,67]
*Artemisia dracunculus* (Common name: Russian tarragon)	essential oils, coumarins, flavonoids, and phenolic acids	↓ systolic blood pressure; ↓ HbA1c and total insulin secretion; ↑ HDL-cholesterol levels	[68]
*Camellia sinensis* (Common name: green tea)	Polyphenols: catechins like EGCG, epigallocatechin,epicatechin-3-gallate and epicatechin	↓ FBG and blood glucose; ↑ insulin sensitivity and secretion; ↓ intestinal glucose absorption by SGLT1 inhibition and oxidative stress; ↑ immune response	[54,55,56,69,70,71]
*Cinnamomum* spp. (Common name: cinnamon)	cinnamaldehyde, procyanidin oligomers	↓ FBG, HbA1c, triglyceride, LDL cholesterol and total cholesterol; ↑ glucose up-take (GLUT4 translocation) and insulin release	[72,73,74]
*Coccinia indica/grandis* (Common name: ivy gourd)	triterpenoid, saponin coccinioside, flavonoid glycoside	↓ levels of the enzymes glucose-6-phosphatase, lactate dehydrogenase; ↑ lipase activity and insulin-secreting through glucose metabolism	[75,76]
*Ipomoea batatas* (Common name: caiapo)	acidic glycoprotein, coumarins, caffeic acid, and flavonoids	↓ FBG and HbA1c; ↑ insulin sensitivity and adiponectin; ↓ fibrinogen levels	[77,78]
*Gymnema sylvestre* (Common name: gurmar)	gymnemic acids, gymnema saponins, and gurmarin dihydroxy gymnemic triacetate	↓ FBG, PPBG and HbA1c of type 2 DM patients; ↑ insulin secretion and C-peptide; ↓ intestinal glucose absorption; ↑ plasma insulin and muscle and liver glycogen in diabetic rats; ↑ islet β cell regeneration	[79,80,81,82]
*Linum ussitatisimum* (Common name: flaxseed)	PUFAs (α-linoleic and linolenic acid), polyphenols, triterpenoids	↓ fasting blood glucose, HbA1c, triglycerides, total and LDL cholesterol, apolipoprotein B; ↑ HDL cholesterol levels	[83,84]
*Momordica charantia* (Common name: bitter melon)	cucurbitane triterpenoids, charantin etc. polypeptide-p	↓ FBG and PPBG levels in type 2 DM; ↓ total cholesterol; ↓ related complications (retinopathy and myocardial infarction); ↑ glucose uptake through stimulation of GLUT-4 translocation, AMPK system; ↓ α-glucosidase activity	[85,86,87,88,89]
*Morus alba* (Common name: morus)	Phenols, flavonoids, anthocyanins, alkaloids	↑ the postprandial glycemic control; ↓ plasma glucose, α-glucosidase; ↑ AMPK and plasma membrane GLUT4 levels in skeletal muscle	[90,91,92]
*Ocimum sanctum* (Common name: holy basil)	tannins and essential oil (eugenol, methyleugenol, and caryophyllene)	↓ FBG and PPBG; ↓ total cholesterol level; ↓ insulin resistance and normalization of serum lipid profile, body weight and BMI, diabetic symptoms, lipid peroxidation; ↑ activity of antioxidant enzymes	[93,94,95,96]
*Opuntia* spp. (Common name: nopal)	flavonoids, phenolic acids, betalains, phytosterol, PUFAs	↓ PPBG and serum insulin, glucose absorption from the intestine and plasma GIP levels; ↑ increase antioxidant activity and glucose uptake (through the AMPK/p38 MAPK signaling pathway and GLUT4 translocation in muscle cells)	[97,98,99]
*Panax ginseng* and *P. quinquefolius* (Common name: Asian and American ginseng)	triterpene saponins, (ginsenosides, protopanaxadiol and protopanaxatriol- type saponins, compound K	↓ FBG and body weight; ↑ glucose metabolism and VEGF expression; ↑ angiogenesis by eNOS activation; ↓ insulin resistance and apoptosis; ↑ fasting serum insulin and insulin sensitivity	[100,101,102]
*Salacia reticulata* (Common name: Kothala himbutu)	polyphenols (mangiferin, catechins, and tannins)	↓ FBG, HbA1c and lipid levels (cholesterol, LDL, VLDL and triglyceride levels)	[103,104,105]
*Silybum marianum* (Common name: milk thistle)	flavonolignans (silymarin complex: silybin and isosilybin, silychristin and silydianin), the flavonol taxifolin	↓ glucose and lipids levels, FBG, HbA1c, total cholesterol, LDL, TG and hepatic enzymes; ↓ PPBG, insulin resistance and insulin production; ↑ antioxidant system (SOD and GPx activities and total antioxidant capacity); ↓ C reactive protein	[106,107,108,109]
*Trigonella foenum graecum* (Common name: fenugreek)	steroid saponins (diosgenin, yamogenin, tigogenin), protoalkaloids, trigonelline, 4-hydroxyisoleucin, soluble fiber fraction	↓ PPBG, FBG, HbA1c, TG, VLDL, lipid; ↓ intestinal glycosidase; ↑ lipogenic enzymes, glucose uptake, HDL level and insulin sensitivity	[110,111]
*Zingiber officinale* (Common name: ginger)	metabolites ginger oleoresin, 8-gingerol, 10-gingerol and 6-shogaol	↓ serum glucose, HbA1c and insulin resistance; ↑ total antioxidant capacity	[112]

Abbreviations: PPBG = Postprandial blood glucose; FBG = Fast blood glucose; AMPK = activating 5-adenosine monophosphate-activated protein kinase; HbA1c = Glycated hemoglobin; TG = Triglyceride; LDL = Light density lipoprotein; HDL = Hight density lipoprotein; PUFAs = Polyunsaturated fatty acids; GIP = glucose-dependent insulinotropic polypeptide; SOD = Superoxide dismutase; GPx = Glutathione peroxidase; eNOS = endothelial nitric oxide synthase; SGLT1 = Sodium glucose transporter protein 1; VEGF = Vascular endothelial growth factor; BMI = Body mass index; ↓ = decrease; ↑ = increase.

## Abbreviations

3-MP: 3-mercaptopyruvate	CD95: Cluster of differentiation 95
ADA: American Diabetes Association	CPSSA: S-allyl-mercapto-captopril
AMS: allyl methyl sulfide	CRP: C-reactive protein
Ang-1: angiopoietin-1	CSE: cystathionine γ-lyase
ARE: antioxidant response elements	CTGF: connective tissue growth factor;
Bcl2: B-cell lymphoma 2	DADS: diallyl disulfide;
BMI: Body mass index	DAS: diallyl monosulfide;
CBS: cystathionine β-synthase	FDPS: Finnish Diabetes Prevention Study
DATES: diallyl tetrasulfide	GIP: Glucose-dependent insulinotropic polypeptide
DATS: diallyl trisulfide	GK: glucokinase
DM: diabetes mellitus	GLUT2: Glucose transporter-2
DPP: Diabetes Prevention Program	GLUT4: Glucose transporter-4
ECs: vascular endothelial cells	GPx: Glutathione peroxidase
ECGC: epigallocatechin-3-gallate	GSH: reduced Glutathione
eNOS: endothelial nitric oxide synthase	GSSH: glutathione persulfide
EPC: endothelial progenitor cell	GST: Glutathione-S-transferase
FBG: Fast blood glucose	H_2_S: Hydrogen sulfide
HbA1c: Glycated hemoglobin	MSNs: mesoporous silica nanoparticles
HDL: Hight density lipoprotein	MST: 3-mercaptopyruvate sulfurtransferase
ICa-L: L-type calcium current	NK cells: Natural killer cells
IK1: inward rectifier potassium current	NO: Nitric oxide
IL-6: Interleukin 6	TGF- β1: transforming growth factor β1
INS-1E: Insulinoma cell line 1E	Nrf2: nuclear factor erythroid 2-related factor 2
IRs: Insulin Receptors	OSCs: organosulfur compounds
LDL: Light density lipoprotein	PUFAs: Polyunsaturated fatty acids
MIN6: mouse insulinoma cell line 6	ROS: Reactive oxygen species
p38 MAPK: p38 mitogen-activated protein kinases	SAC: S-allyl cysteine
PFM: polylactic fibrous membranes	SAMC: S-allylmercaptocysteine
PLP: pyridoxal 5’-phosphate	SAMG: S-allylmercaptoglutatione
PPARs: peroxisome proliferator-activated receptors	TNF-α: Tumor necrosis factor
PPBG: Postprandial blood glucose	VEGF: Vascular endothelial growth factor
SGLT1: Sodium glucose transporter protein 1	VLDL: Very low density lipoprotein
SOD: Superoxide dismutase	WHO: World Health Organization.
TG: Triglyceride	
AMPK: activating 5-adenosine monophosphate-activated protein kinase	
P13k/Akt: phosphoinositide-3-kinase/ Protein Kinase B	
p-ERK1/2: phosphorylated extracellular signal–regulated kinases 1/2	
NF-κB: nuclear factor kappa light chain enhancer of activated B cells	
EASD: European Association for the Study of Diabetes	
NOX: nicotinamide adenine dinucleotide phosphate oxidase	
HIT-T15: insulin release from a cloned hamster B-cell line	
